# Autophagy is activated and involved in cell death with participation of cathepsins during stress-induced microspore embryogenesis in barley

**DOI:** 10.1093/jxb/erx455

**Published:** 2018-01-04

**Authors:** Ivett Bárány, Eduardo Berenguer, María-Teresa Solís, Yolanda Pérez-Pérez, M Estrella Santamaría, José Luis Crespo, María C Risueño, Isabel Díaz, Pilar S Testillano

**Affiliations:** 1 Biological Research Center, CIB, Madrid, Spain; 2 Center of Plant Biotechnology and Genomics, CBGP, UPM, Madrid, Spain; 3 Institute of Plant Biochemistry and Photosynthesis, IBVF, CSIC, Seville, Spain; 10 University of Nottingham, UK

**Keywords:** Autophagy, barley, caspase-like activity, cathepsins, cell death, cysteine C1A proteases, microspore embryogenesis, ROS, stress response

## Abstract

Microspores are reprogrammed towards embryogenesis by stress. Many microspores die after this stress, limiting the efficiency of microspore embryogenesis. Autophagy is a degradation pathway that plays critical roles in stress response and cell death. In animals, cathepsins have an integral role in autophagy by degrading autophagic material; less is known in plants. Plant cathepsins are papain-like C1A cysteine proteases involved in many physiological processes, including programmed cell death. We have analysed the involvement of autophagy in cell death, in relation to cathepsin activation, during stress-induced microspore embryogenesis in *Hordeum vulgare*. After stress, reactive oxygen species (ROS) and cell death increased and autophagy was activated, including *HvATG5* and *HvATG6* up-regulation and increase of ATG5, ATG8, and autophagosomes. Concomitantly, cathepsin L/F-, B-, and H-like activities were induced, cathepsin-like genes *HvPap-1* and *HvPap-6* were up-regulated, and HvPap-1, HvPap-6, and HvPap-19 proteins increased and localized in the cytoplasm, resembling autophagy structures. Inhibitors of autophagy and cysteine proteases reduced cell death and promoted embryogenesis. The findings reveal a role for autophagy in stress-induced cell death during microspore embryogenesis, and the participation of cathepsins. Similar patterns of activation, expression, and localization suggest a possible connection between cathepsins and autophagy. The results open up new possibilities to enhance microspore embryogenesis efficiency with autophagy and/or cysteine protease modulators.

## Teaser

Autophagy is activated and plays a role in stress-induced cell death during microspore embryogenesis, along with cathepsin-like C1A protease activities. Autophagy inhibition reduces cell death, improving embryogenesis induction efficiency.

## Introduction

Plant cell plasticity and ability to regenerate embryos in *in vitro* culture have been extensively exploited for decades, in the areas of plant propagation, breeding, and conservation of genetic resources (Germaná and Lambardi, 2016). *In vitro* embryogenesis is a fascinating system to study cellular reprogramming and acquisition of totipotency, as well as an alternative to study early embryogenesis events since zygotes and immature embryos produced *in planta* are surrounded by maternal tissues and are difficult to analyse. Microspore embryogenesis is an *in vitro* system in which the haploid microspore is reprogrammed by the application of external stress treatment and enters into an embryogenesis pathway (Bárány *et al.*, 2005; Prem *et al.*, 2012). The resulting haploid and double-haploid embryos and generated plants are important biotechnological tools in plant breeding for the rapid generation of isogenic new varieties as they represent a source of new genetic variability, fixed in complete homozygous plants and obtained in only one generation step (Maluszynski et al., 2003). Despite the usefulness of stress-induced *in vitro* embryogenesis in breeding programmes, the efficiency of the system in many species of economic interest is still limited since it is greatly affected by many factors (El-Tantawy *et al.*, 2013; Rodríguez-Sanz *et al.*, 2014, 2015; Solís *et al.*, 2012, 2015, 2016; Testillano *et al.*, 2010), and primarily by the occurrence of cell death induced by the stress applied to trigger embryogenesis. In barley, microspore embryogenesis is efficiently induced by cold stress treatment in isolated microspore cultures (Rodríguez-Serrano *et al.*, 2012).

Autophagy is a universal degradation pathway in all eukaryotes, including plants, that recycles cell materials upon stress conditions or during specific developmental processes, thereby promoting cell survival (reviewed in Hofius *et al.*, 2017; Masclaux-Daubresse *et al.*, 2017). In addition to this survival role, autophagy can also play critical roles as a cell death initiator and/or executioner. Increasing evidence indicates the involvement of autophagy in plant cell death (Minina *et al.*, 2013, 2014*b*; Yang and Bassham, 2015). In *Picea abies* embryos, it has been demonstrated that autophagy is responsible for cell self-disassembly during programmed cell death (PCD) (Minina *et al.*, 2013). In plants, autophagy-directed degradation of cellular components occurs mainly in vacuoles. It is initiated by the engulfment of subcellular components into a double membrane structure, the autophagosome, the outer membrane of which further fuses with the vacuole membrane, the tonoplast. This results in the release of the so-called autophagic bodies (single-membrane structures containing the cargo) at the vacuole interior where degradation takes place via the activity of lytic enzymes. In different plant species, autophagosomes either can fuse directly with the central vacuole or can first fuse with a smaller vacuole or lysosome-like organelle, which begins content degradation (Bassham, 2007). Activation of autophagy involves the induction of AuTophaGy-related ATG genes and activation of specific proteases. In the case of barley, 25 ATG genes have been characterized (Avila-Ospina *et al.*, 2016; Masclaux-Daubresse *et al.*, 2017). Among them, ATG5, ATG6, and ATG8 proteins play crucial roles in autophagosome formation (Li and Vierstra, 2012; Michaeli *et al.*, 2016).

Plant cells actively produce reactive oxygen species (ROS) at low levels but, as a response to stress, cell production of ROS increases. ROS act as signalling molecules to control processes such as PCD and stress response. Excessive ROS levels may cause irreversible oxidative damage and activate signalling pathways ultimately leading to cell death (Apel and Hirt, 2004). Recent studies in plants and algae have described the activation of autophagy in response to several stress conditions that increase ROS production (Liu and Bassham, 2012; Pérez-Pérez *et al.*, 2010, 2012). These findings suggest a strong link between autophagy and ROS production in plants.

Autophagy and cell death proteases are well characterized in animals, and caspases are thought to be the major proteases involved. Although to date no functional homologues of animal caspases have been identified in plants, several indirect pieces of evidence suggest the existence of functionally related proteases with similar substrate specificity. The involvement of caspase-3-like enzymatic activity in plant PCD has been well documented, and its specific inhibitors block completion of PCD (Bonneau *et al.*, 2008; Solís *et al.*, 2014), although the identity of the protease(s) responsible has not yet been fully resolved. Recently, it has been reported that caspase-3 inhibitors reduce PCD in Arabidopsis by targeting cathepsin B, another major plant protease involved in PCD (Ge *et al.*, 2016).

Cathepsins are papain-like C1A cysteine proteases, as classified in the MEROPS peptidase database (Rawlings *et al.*, 2016). They are well known lysosomal proteases with a role in autophagy and cell death, in animals (Turk and Stoka, 2007). It is well documented in animals that cathepsins are responsible for driving proteolytic degradation in lysosomes and have a critical role in the terminal degradation of proteins within autolysosomes, following the autophagosome fusion (Jung *et al.*, 2015; Kroemer and Jäättelä, 2005; Man and Kanneganti, 2016). In plants, this enzyme group with proteolytic activity is involved in many physiological processes such as senescence, abscission, fruit ripening, and PCD, and in the mobilization of proteins accumulated in seeds and tubers Martínez *et al.*, 2012; Díaz-Mendoza et al., 2014, 2016). Moreover, C1A proteases actively participate in proteolysis induced by biotic and abiotic stresses (Díaz-Mendoza *et al.*, 2014; Velasco-Arroyo *et al.*, 2016), but less is known about the possible role of plant cathepsins in autophagosome degradation. Plant cathepsins are grouped as cathepsin L-, B-, H-, and F-like according to their gene features and phylogenetic relationship. The activity of these proteases is regulated by specific inhibitors, termed phytocystatins (Martínez et al., 2009; Díaz-Mendoza *et al.*, 2016), and is also the target of synthetic exogenous inhibitors such as E-64. In barley, both C1A proteases and cystatins have been studied in depth (Martínez *et al.*, 2009, 2012; Díaz-Mendoza *et al.*, 2014, 2016). However, there is no information on the action of these proteases during stress-induced microspore embryogenesis.

During stress-induced microspore embryogenesis, we have reported increasing cell death levels and caspase-3-like activity, after the inductive stress to trigger microspore reprogramming, in barley (Rodríguez-Serrano *et al.*, 2012). Nevertheless, prior to the present work, no studies had been developed on the participation of autophagy during the induction of microspore embryogenesis mediated by stress in barley.

In this work, we have studied the involvement of autophagy in cell death occurrence during stress-induced microspore embryogenesis of barley, in relation to cathepsin activation. The results indicated that autophagy is induced in microspores after the inductive stress. Concomitantly, cathepsins are also activated and show similar patterns of expression and localization to ATGs. Inhibition of autophagy and cathepsins reduced cell death levels and increased the embryogenesis induction rate. Taken together, the results indicate a role for autophagy in cell death at early stages of stress-induced microspore embryogenesis, a death process in which cathepsins also participate.

## Materials and methods

### Plant material and *in vitro* microspore embryogenesis culture

Winter barley cultivars (*Hordeum vulgare* L. cv. Igri) were used as donor plants. Seeds were vernalized in soil for 1 month at 4 °C, grown at 12 °C with a 12 h light/12 h dark photoperiod (10 000–16 000 lux) for 1 month in a growth chamber (Sanyo MLR-351-H, relative humidity 70%), and then grown in a greenhouse under a controlled temperature of 18 °C. *In vitro* cultures of isolated microspores, the most responsive stage for embryogenesis induction, were performed by stress treatment of 4 °C, as previously described (Rodríguez-Serrano *et al.*, 2012).

### Cell death detection

Microspore culture samples were incubated with a 0.25% (w/v) aqueous solution of Evans Blue for 30 min and observed with a light microscope under bright field. Mean percentages of dead cells (stained by Evans Blue) were quantified in random micrographs from two replicates of three independent experiments, as described (Solís *et al.*, 2015).

### ROS detection

Microspore culture samples were incubated for 1 h, in the dark, with 10 µM dihydroethidium (DHE) to detect ROS, specifically superoxide radicals (Rodríguez-Serrano *et al.*, 2012). As negative control, samples were incubated for 1 h before DHE with 4 mM MnCl_2_, an O^2–^ scavenger. After washing, samples were immediately observed with a confocal microscope (Leica TCS SP5) and signal captured as red ﬂuorescence (490 nm excitation; 520 nm emission).

### Treatments with inhibitors

At the time of culture initiation, several inhibitors (Table 1) were added to the microspore culture plates. Ac-DEVD-CHO, E-64, and concanamycin A (ConA) were added from concentrated stock solutions dissolved in ethanol (E-64) and DMSO (Ac-DEVD-CHO and ConA). Controls of solvent effects were performed by adding the same volumes of ethanol or DMSO to untreated cultures. 3-Methyladenine (3-MA) and MnCl_2_ were directly dissolved in the culture medium. Short treatments were carried out from culture initiation during 4 d. Mean percentages of ‘proembryos’ were quantified from random samples of three independent experiments, as previously described (Berenguer *et al.*, 2017).

**Table 1.** Inhibitors and conditions used for *in vitro* treatments of microspore cultures

Name	Concentration	Brand
MnCl_2_	4 mM	Merck
Ac-DEVD-CHO	5 µM	Sigma
3-MA	5 mM	Sigma
E-64	1µM	Sigma
ConA	1 µM	Sigma

MnCl_2_, manganese chloride; Ac-DEVD-CHO, *N*-acetyl-l-α-aspartyl-l-α-glutamyl-*N*-(2-carboxyl-1-formylethyl)-l-valinamide; 3-MA, 3-methyladenine; E-64, *trans*-epoxysuccinyl-l-leucylamido(4-guanidino)butane; ConA, concanamycin A.

### Antibodies

Cathepsin antibodies against HvPap-1, HvPap-6, and Hv-Pap-19 proteases were produced in rabbits by Pineda Antibody Services (Berlin, Germany), against specific peptide sequences of each protease (Supplementary Table S1 at *JXB* online); they were previously produced and reported to recognize these proteases in barley leaves (Díaz-Mendoza *et al.*, 2016). ATG5 antibody was kindly provided by Dr M.F. Suárez (University of Málaga, Spain), produced in rabbits with purified recombinant ATG5 protein of spruce as antigen (Supplementary Method S1). ATG8 antibody was produced in the laboratory of Dr J.L. Crespo (IBFV, Seville, Spain), and was reported to recognize specifically Arabidopsis ATG8 (Álvarez *et al.*, 2012; Pérez-Pérez *et al.*, 2010).

### Fixation and processing for light and electron microscopy analyses

For light microscopy, *in vitro* samples were collected and fixed in 4% paraformaldehyde in phosphate-buffered saline (PBS), pH 6.8, overnight at 4 °C. Samples were dehydrated in acetone and embedded in Technovit 8100 resin (Kulzer, Germany) at 4 °C. Semi-thin resin sections were either stained with toluidine blue and observed under bright field, for structural analysis, or stored at 4 °C and used for immunofluorescence (Solís *et al.*, 2016). For electron microscopy, samples were fixed in Karnowsky fixative (4% paraformaldehyde, 5% glutaraldehyde in 0.025 M cacodylate buffer with 0.5 mg ml^–1^ calcium chloride) for 4 h at room temperature, post-fixed in 2% osmium tetroxide, dehydrated in an ethanol series and propylene oxide, and embedded in Epon 812 resin. Ultrathin sections were counterstained by uranyl acetate and lead citrate, and examined in an electron microscope (JEOL JEM 2100).

### Immunofluorescence and confocal microscopy

Semi-thin sections were blocked by 5% (w/v) BSA and incubated for 1 h with the corresponding primary polyclonal antibody diluted in 1% BSA at 1:100 (HvPap-1), 1:50 (HvPap-6), 1:20 (HvPap-19), and 1:50 (ATG5 and ATG8). After washing in PBS, signal was revealed with Alexa Fluor 488-labelled anti-rabbit IgG antibody (Molecular Probes) diluted 1:25 in 1% BSA for 45 min in darkness. Finally, sections were counterstained with 1 mg ml^−1^ DAPI for 10 min and analysed by confocal laser microscopy (Leica TCS-SP5-AOBS, Vienna, Austria). Images of maximum projections were obtained with software of the confocal microscope (Leica software LCS version 2.5). Negative controls were performed avoiding the primary antibody.

### MDC *in vivo* staining

Microspore samples from untreated and inhibitor-treated cultures were stained with 0.05 mM monodansylcadaverine (MDC; Sigma-Aldrich), at room temperature for 30 min in darkness (Contento *et al.*, 2005). After incubation, cells were washed twice with PBS and immediately observed by confocal microscopy (Leica TCS SP5). Fluorescence of intracellular MDC was observed selecting wavelengths of 405 nm for excitation and 435–483 nm for emission.

### Quantitative real-time PCR analysis (RT-qPCR)

Total RNA was extracted from *in vitro* samples using the RNeasy^®^ Plant Micro and RNeasy^®^ Plant Mini kits (Qiagen) according to the manufacturer’s instruction. cDNAs were obtained from 2 µg of RNA using the Superscript™ II reverse transcriptase (Invitrogen) according to Solís *et al.* (2012). RT-qPCR analyses were performed using the SsoAdvanced™ Universal SYBR^®^Green Supermix on the iQ™5 Real-Time PCR Detection Sytem (Biorad). The oligonucleotides used are described in Supplementary Table S2, and qPCR conditions were as previously described (Berenguer *et al.*, 2017). All qPCRs were run in duplicate, and the *Cyclophilin* gene was used as the internal reference gene. Transcript levels were normalized to the vacuolated microspore levels. Data were analysed with the Bio-Rad CFX Manager 3.0 (3.01224.1015) (Biorad), using the Livak calculation method (Livak and Schmittgen, 2001).

### Protein quantification and protease activities

Total soluble proteins were extracted from *in vitro* samples according to Velasco-Arroyo *et al.* (2016), using the method of Bradford (1976) for protein quantification. Enzymatic activity assays were performed as previously described (Velasco-Arroyo *et al.*, 2016) with minor modifications. Cathepsin L-/F-, B-, and H-like activities were assayed using Z-FR-AMC (*N*-carbobenzoxy-Phe-Arg-AMC), Z-RR-AMC (*N*-carbobenzoxy-Arg-Arg-AMC), and Bz-FVR-AMC (Bz-Phe-Val-Arg-AMC) substrates, respectively. The reaction was incubated at 28 °C for 1 h. All assays were carried out in duplicate. Blanks were used to account for spontaneous breakdown of substrates, and the results were expressed as nmol of hydrolysed substrate per mg of protein per min (nmol min^–1^ mg^–1^). The system was calibrated with known amounts of AMC (7-amino-4-methylcoumarin) in a standard reaction mixture.

### Immunoblot analysis

Protein extracts were prepared from *in vitro* cultures by grinding microspore embryos in liquid nitrogen before the addition of 100 μl of extraction buffer (150 mM NaCl, 50 mM sodium phosphate, pH 6.0, and 2 mM EDTA). Extracted proteins were quantified by the Bradford method (Bradford, 1976) with BSA as standard. Proteins were separated on SDS–polyacrylamide gels (12%,w/v), electro-transferred onto nitrocellulose membranes, and blocked in 3% BSA containing 0.5% Tween-20 for 1 h. Immunoblotting was performed according to Velasco-Arroyo *et al.* (2016) with the polyclonal antibodies to cathepsins, at 1:2500, 1:2500, and 1:5000 dilutions for HvPap-1, HvPap-6, and HvPap-12 respectively. Peroxidase-conjugated anti-rabbit IgG (Sigma), diluted at 1:20 000 (v/v), was used as a secondary antibody for detection with ECL Plus.

### Data analysis

Statistical differences among several stages were tested by one-way ANOVA followed by Tukey’s multiple comparison tests. Significant differences between untreated and treated cultures were tested by Student’s *t*-test, in all cases with *P*≤0.05.

## Results

### Cell death occurrence, ROS production, and caspase-3-like proteolytic activity in stress-induced microspore embryogenesis

**Fig. 1.**
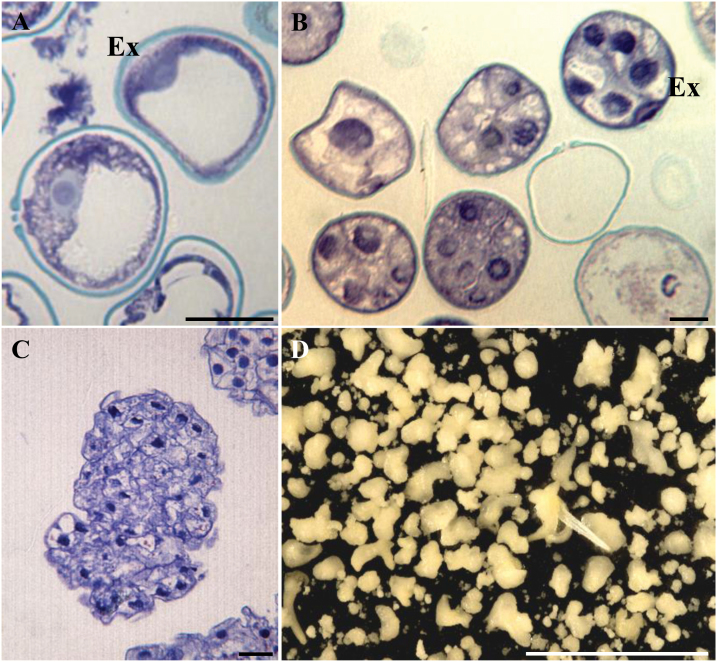
Stress-induced microspore embryogenesis in *Hordeum vulgare*. Micrographs of toluidine blue-stained semi-thin sections for general structural analysis. (A) Vacuolated microspore at culture initiation. (B) Proembryos on microspore culture 4 d after stress, still surrounded by the exine. (C) Early transitional embryo. (D) Microspore-derived embryos after 30 d in culture observed under the stereomicroscope. Ex, exine. Scale bars represent in (A–C) 20 µm, in (D) 10 mm.

Microspore embryogenesis was induced by cold stress (4 °C) in barley using isolated microspore *in vitro* cultures, as previously reported (Rodríguez-Serrano *et al.*, 2012). Vacuolated microspores (Fig. 1A), the most responsive stage for embryogenesis induction, were subjected to the inductive stress treatment. Four days after induction and culture initiation, multicellular structures still surrounded by the exine, the so-called microspore-derived ‘proembryos’, were produced (Fig. 1B). In 4 d cultures, proembryos were accompanied by non-responsive and dead microspores (Fig. 1B). During the following days of culture, microspore embryogenesis progressed, the exine broke down, and embryos developed (Fig. 1C) and followed a pathway similar to zygotic embryogenesis in monocot species, producing transitional and scutellar embryos and then, after 30 d in culture, coleoptilar embryos (Fig. 1D).

**Fig. 2.**
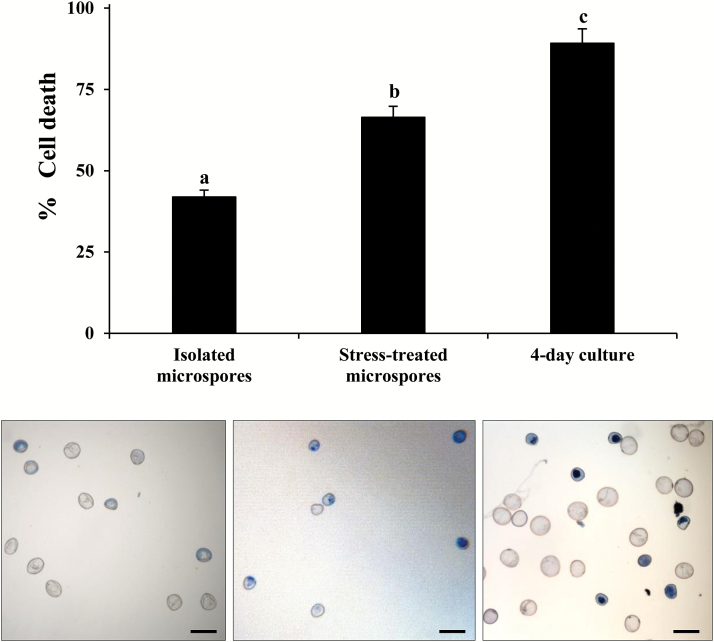
Cell death in stress-induced microspore embryogenesis. Histogram showing the percentage of cell death after cell isolation and inductive stress identified by Evan’s blue staining. Micrographs showing dead microspores as blue cells after Evan’s blue staining. Bars in columns indicate the SE. Scale bars in micrographs represent 60 µm. Different letters on columns indicate significant differences among stages, according to ANOVA and Tukey’s tests at *P*<0.05.

The percentage of dead cells, identified by positive Evans blue staining (Fig. 2), was quantified at several culture steps: ‘isolated microspores’ (microspores extracted from spikes but not treated by stress), ‘stress-treated microspores’ (isolated microspores after the inductive stress), and ‘4 d cultures’ (stage of formation of the proembryo). Results showed the occurrence of cell death in isolated microspores, probably due to the isolation procedure and the presence of dead cells in the spike. Dead cell levels significantly increased (*P*<0.05, ANOVA and Tukey’s tests) after the stress treatment in stress-treated microspores and in 4 d cultures (Fig. 2).

**Fig. 3.**
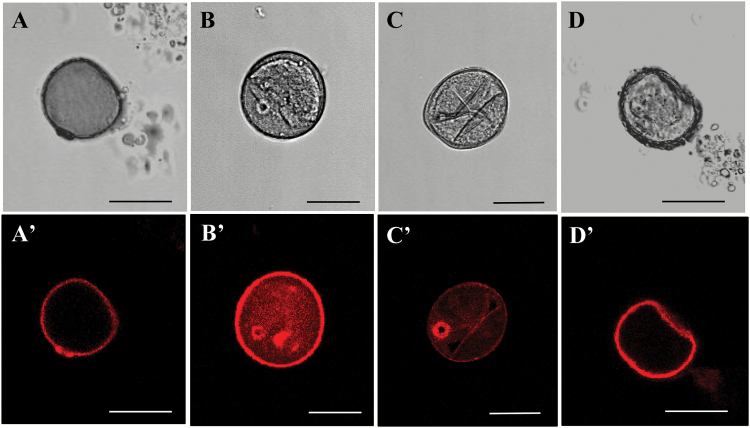
ROS staining during stress-induced microspore embryogenesis. Specific staining with dihydroethidium (DHE). Confocal laser scanning microscopy analysis of (A, A') isolated microspore, (B, B') stress-treated microspore, (C, C') 4 d culture proembryo, (D, D') stress-treated microspore after incubation with MnCl_2_ (O_2_^–^ scavenger). Scale bars represent 25 µm.

**Fig. 4.**
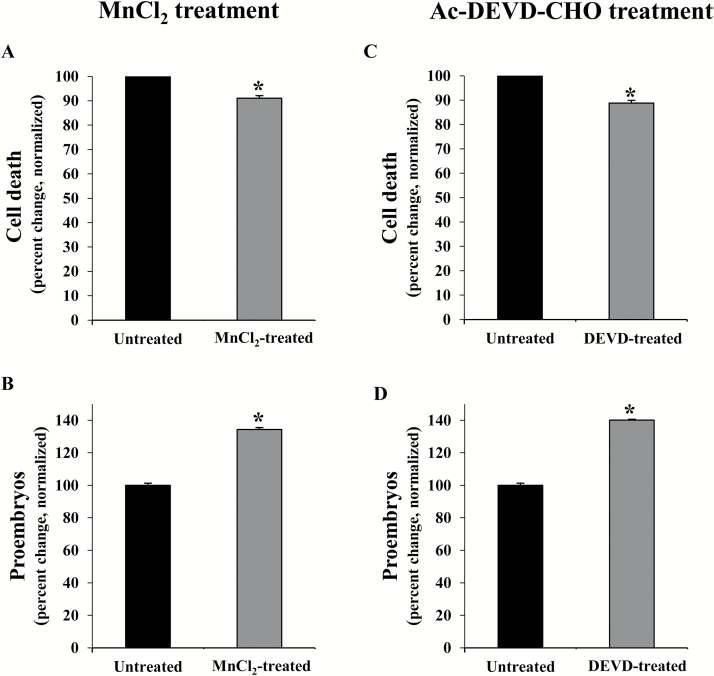
Effect of treatments with MnCl_2_ (O_2_^–^ scavenger) and Ac-DEVD-CHO (caspase-3 inhibitor) in stress-induced microspore embryogenesis. (A, C) Quantification of cell death levels, identified by Evan’s blue staining, 4 d after stress in untreated microspore cultures and cultures treated with MnCl_2_ (A) and Ac-DEVD-CHO (C). (B, D) Quantification of proembryos (as an indicator of microspore embryogenesis initiation) in microspore cultures, 4 d after stress, treated with MnCl_2_ (B) and Ac-DEVD-CHO (D). In all histograms, results are expressed as percentages (percent change) and referred to the mean percentage of dead cells or proembryos in control cultures which has been normalized to 100%. Bars indicate the SE. Asterisks indicate significant differences between treated and untreated cultures, within each treatment, assessed by Student’s *t*-test, at *P*<0.05.

ROS production was analysed by specific staining with the fluorescence probe DHE which speciﬁcally reacts with the intracellular superoxide anion (O_2_^−^) *in vivo* (Zhao *et al.*, 2003). Under confocal microscopy analysis, almost no signal was detected in isolated microspores (Fig. 3A) or 4 d cultures (Fig. 3C), whereas an intense fluorescence was observed in many stress-treated microspores (Fig. 3B). If stress-treated microspores were incubated with the ROS scavenger MnCl_2_, DHE staining did not provide any signal (Fig. 3D), confirming the specificity of the probe for ROS. The exine showed unspecific autofluorescence of different intensities in all cases (Fig. 3A'–D').

To evaluate the effect on cell death of the elimination of ROS in stress-treated microspore cultures, treatments were performed with the ROS scavenger MnCl_2_, which specifically scavenges superoxide anions. Quantification of dead cells, by Evans blue staining, was carried out in MnCl_2_-treated and untreated cultures 4 d after the inductive stress. Results showed a significant reduction (*P*<0.05, Student’s *t*-test) in cell death levels in microspore cultures treated with the ROS scavenger in comparison with untreated cultures (Fig. 4A), indicating the involvement of ROS in cell death. As a consequence, the proportion of proembryos formed in microspore cultures treated with the ROS scavenger was significantly higher (*P*<0.05, Student’s *t*-test) than in control cultures (Fig. 4B).

We have previously reported the induction of caspase-3-like proteolytic activity in barley microspore cultures after the inductive stress (Rodríguez-Serrano *et al.*, 2012). In the present study, to analyse the role of this enzymatic activity, treatments with Ac-DEVD-CHO, a specific inhibitor of caspase-3 activity, were performed in stress-treated microspores. The effects of the treatment with Ac-DEVD-CHO on cell death and embryogenesis induction were evaluated in control and treated cultures. The proportion of dead cells after the inductive stress was significantly lower (*P*<0.05, Student’s *t*-test) in microspore cultures treated with the inhibitor than in control cultures (Fig. 4C). After 4 d in culture, the number of proembryos was determined as an indicator of initiation of microspore embryogenesis. The results showed a statistically significant increase (*P*<0.05 Student’s *t*-test) in the proportion of proembryos produced in microspore cultures treated with the caspase-3 inhibitor in comparison with control cultures (Fig. 4D), probably as a consequence of the reduction in cell death.

### ATG gene expression and protein localization in stress-induced microspore embryogenesis

**Fig. 5.**
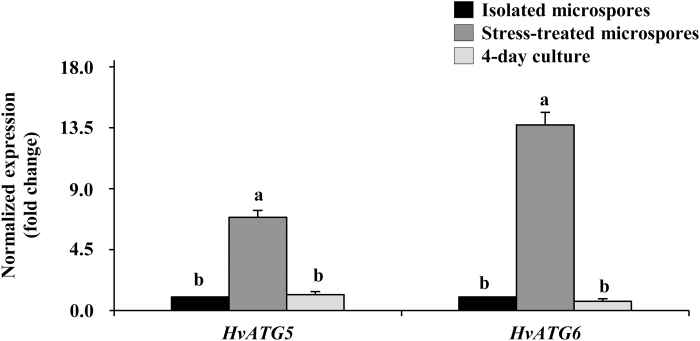
Gene expression patterns of autophagy genes *HvATG5* and *HvATG6* during stress-induced microspore embryogenesis. Histogram showing relative changes of mRNA levels normalized to isolated microspore levels, as determined by RT-qPCR. Bars indicate the SE. Different letters indicate significant differences among stages within the expression of each gene according to ANOVA and Tukey’s tests at *P*<0.05.

Among the 25 ATG genes characterized in barley (Avila-Ospina *et al.*, 2016; Masclaux-Daubresse *et al.*, 2017), to evaluate the possible activation of autophagy in microspore embryogenesis cultures after the inductive stress, expression analyses were conducted for two key autophagy genes, *HvATG5* and *HvATG6*, identified in barley with only one gene isoform each (Avila-Ospina *et al.*, 2016). RT-qPCRs showed similar expression patterns for both ATG genes: a low level of expression in isolated microspores, before the stress, and high gene expression in stress-treated microspores (Fig. 5). Later, in 4 d microspore cultures, expression decreased, dropping to levels similar to those seen with isolated microspores (Fig. 5).

**Fig. 6.**
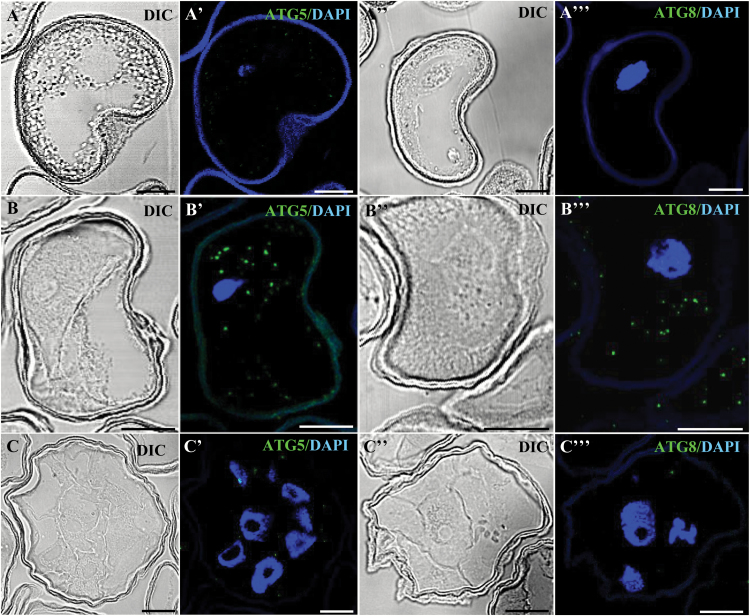
Immunolocalization of autophagy proteins HvATG5 and HvATG8 during stress-induced microspore embryogenesis. Immunofluorescence and confocal laser scanning microscopy analysis of isolated microspore (A–A'''), stress-treated microspore (B–B'''), and 4 d culture proembryo (C–C'''). (A–C, A''–C'') Normarsky’s differential interference contrast (DIC). (A'–C', A'''–C''') Merged images of ATG immunofluorescence (green) and DAPI staining of nuclei (blue). (A'–C') HvATG5. (A'''–C''') HvATG8. Scale bars represent in (A–B'') 10 µm, in (C–C'') 20 µm.

ATG5 and ATG8 proteins, which have a crucial role in autophagy (Bassham, 2009; Li and Vierstra, 2012; Michaeli *et al.*, 2016), were localized by using specific antibodies. Immunofluorescence assays and confocal analyses on semi-thin sections showed no labelling in isolated microspores with either ATG5 or ATG8 antibodies (Fig. 6A, A', A''), whereas in stress-treated microspores ATG5 and ATG8 localized in small punctuate cytoplasmic structures (Fig. 6B, B', B''). Four days after the stress, no significant immunofluorescence labelling was observed with either of these two autophagy antibodies (Fig. 6C, C', C'').

### Effects of treatments with the inhibitors of autophagy 3-MA, E-64, and Con A on cell death occurrence and microspore embryogenesis initiation

Functional analyses of autophagy in microspore embryogenesis were performed by treating microspore cultures *in vitro* with 3-MA, E-64, and ConA, three drugs commonly used to inhibit autophagy in plants (Matsuoka *et al.*, 1997; Takatsuka *et al.*, 2004; Sláviková *et al.*, 2005; Bassham, 2009, 2015; Merkulova *et al.*, 2014; Shin *et al.*, 2014; Yano *et al.*, 2015).

**Fig. 7.**
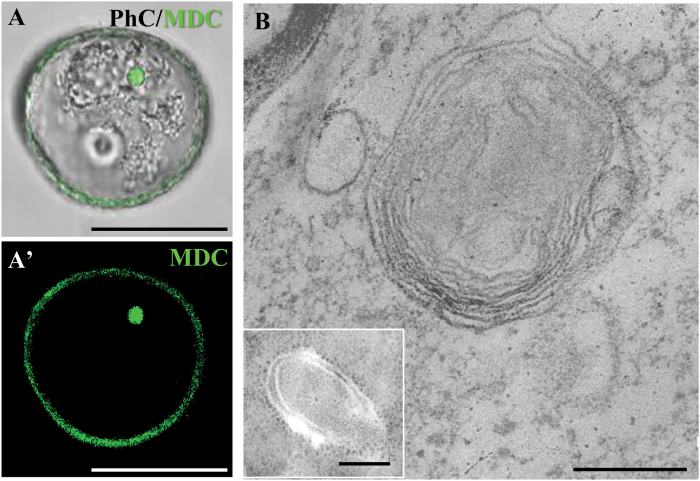
Detection of autophagosomes and autophagic bodies in stress-treated microspores by monodansylcadaverine (MDC) staining and ultrastructural analysis. (A, A') MDC staining of an autophagosome/autophagic body (green) under confocal microscopy, (A) merged DIC and fluorescence image. (B) Electron microscopy images of autophagosomes. The main micrograph shows an advanced/mature autophagosome that has engulfed cytoplasmic organelles/material. The inset shows an autophagosome at an early stage of its formation. Bars represent in (A, A') 25 µm, in (B) 0.5 µm, in (inset) 0.2 µm.

Autophagosomes, autolysosome-like structures, and autophagic bodies can be detected by *in vivo* MDC staining in plant cell suspensions (Niemann *et al.*, 2000; Contento *et al.*, 2005). Microspores were stained by MDC and analysed by confocal microscopy. Stress-treated microspores showed strong MDC fluorescence on small spherical cytoplasmic spots (Fig. 7A, A'). These spots were of different sizes, and were occasionally observed within vacuoles, and, therefore, they most probably corresponded to autophagosomes and autophagic bodies. Electron microscopy provided evidence of autophagic structures in stress-treated microspores. Ultrastructural analysis revealed the presence of early and mature autophagosomes. Autophagosomes at an early stage of their formation appeared as double-membrane structures with semi-dense content, similar to the cytoplasm (Fig. 7B, inset). Advanced/mature autophagosomes that had engulfed cytoplasmic structures/organelles showed double- and multiple-membrane structures, with organelle and membrane remnants in their interior (Fig. 7B).

**Fig. 8.**
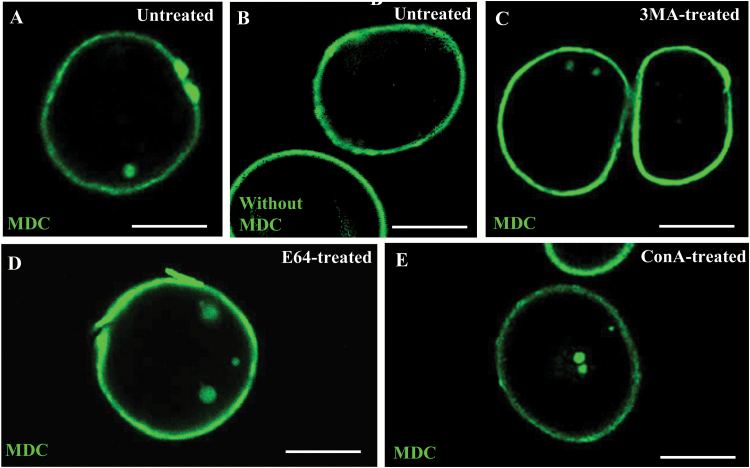
Effects of treatments with 3-MA, E-64, and ConA on autophagosome presence in microspore cultures. (A, C–E) MDC staining and confocal laser scanning microscopy analysis of stress-treated microspores. (B) Control without MDC in stress-treated microspores, which show unspecific autofluorescence of the microspore wall, the exine. (A) Untreated microspore culture. (C) Microspore culture treated with 3-MA. (D) Microspore culture treated with E-64. (E) Microspore culture treated with ConA. Scale bars in micrographs represent 20 µm.

**Fig. 9.**
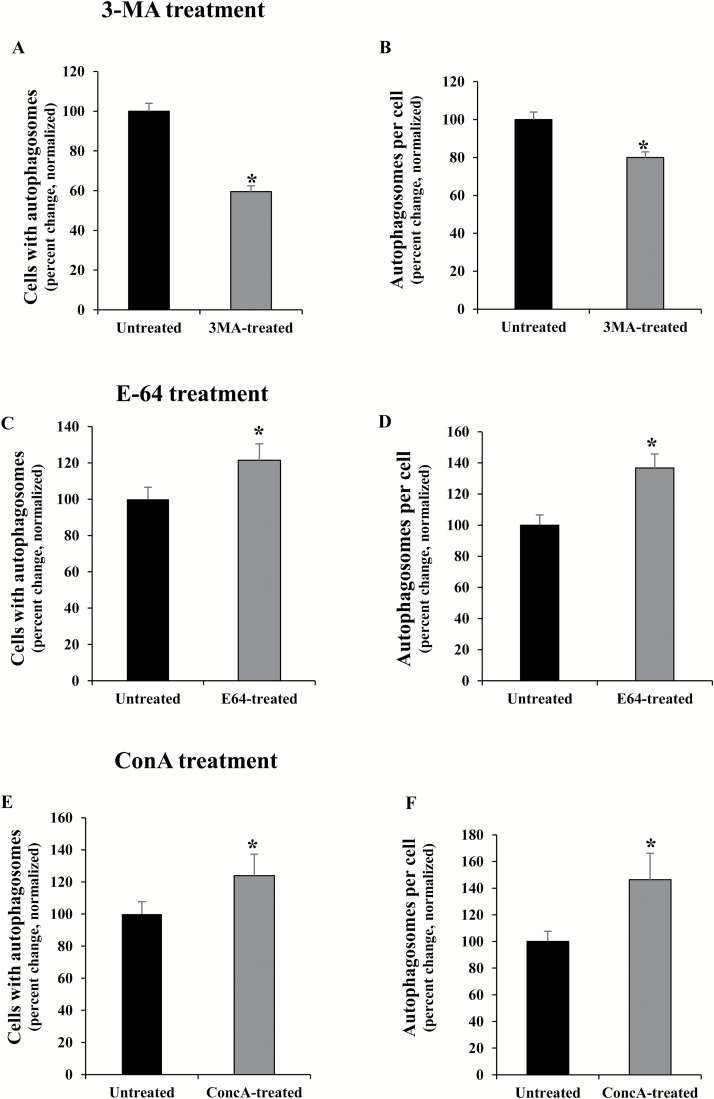
Quantitative analyses of autophagy in microspore cultures after treatments with autophagy inhibitors 3-MA, E64, and ConA. (A, B) 3-MA treatment. (C, D) E-64 treatment. (E, F) ConA treatment. (A, C, E) Cells with autophagosomes (MDC-positive cells) in untreated and treated microspore cultures. (B, D, F) Autophagosomes per cell in untreated and treated microspore cultures. In all histograms, results are expressed as percentages (percent change) and referred to the mean percentage in untreated cultures which has been normalized to 100%. Bars in histograms indicate the SE. Asterisks indicate significant differences between treated and untreated cultures, within each treatment, assessed by Student’s *t*-test, at *P*<0.05.

3-MA is an inhibitor of phosphatidylinositol 3-kinase (PtdIns3K) involved in the formation of the autophagosome (Li and Vierstra, 2012). 3-MA has been reported to block autophagosome formation in tobacco BY2 culture cells, at 5 mM concentration (Takatsuka *et al.*, 2004). To evaluate its effects on microspore cultures, 5 mM 3-MA was added to the culture media of stress-treated microspores. Inhibition of autophagy by 3-MA was measured in microspores after the inductive stress by quantifying the autophagosomes and autophagic bodies, as revealed by MDC, in untreated and 3-MA-treated cultures (Fig. 8A, C). Control assays without MDC staining in stress-treated microspores did not provide fluorescence to any subcellular structure, except for the microspore wall, the exine, which exhibited unspecific autofluorescence in all microspores (Fig. 8B). 3-MA-treated and untreated cultures showed cells with MDC-stained spots (one or two) and cells without any stained structures (Fig. 8A, C). The results of the quantification showed a significant reduction of autophagy in microspores treated with 3-MA in comparison with control cultures (Student’s *t*-test, *P*<0.05), as revealed by the reduction in the proportion of cells with autophagosomes (Fig. 9A) and the decrease in the mean number of autophagosomes per cell (Fig. 9B).

The cysteine protease inhibitor E-64 was also added to microspore cultures after the inductive stress to trigger embryogenesis. In many plant species, E-64 has been reported to block autophagy at the step of autophagosome degradation, which therefore leads to the accumulation of autophagic bodies in vacuoles or smaller autolysosome-like organelles in the cytoplasm (Bassham, 2007, 2015; Moriyasu and Inoue, 2008). Microspore cultures treated with E-64 showed cells with higher numbers of MDC-stained spots in their cytoplasms (up to 4–6 spots) compared with untreated cultures, whose cells showed 0–2 spots per cell (Fig. 8A, D). Moreover, quantitative analyses showed a significantly higher proportion of cells with autophagic bodies (Fig. 9C), as well as a significant increase in the mean number of autophagic structures per cell (Fig. 9D) in E-64-treated cultures compared with untreated cultures (Student’s *t*-test, *P*<0.05).

ConA, which inhibits vacuolar proton pumps and leads to increased vacuolar pH and inactivation of acid hydrolases, has been used to inhibit autophagic body degradation and to assess autophagic flux in plant tissues and cell suspensions (Matsuoka *et al.*, 1997; Sláviková *et al.*, 2005; Shin *et al.*, 2014; Bassham, 2015; Yano *et al.*, 2015). ConA treatment of microspore cultures showed a similar effect to E-64 treatment. In ConA-treated cultures, MDC staining revealed cells with a higher number of autophagosomes (3–6) than untreated cultures (Fig. 8A, E). The quantitative analyses of MDC-positive spots showed that ConA-treated cultures presented a significant increase in both the proportion of cells with autophagosomes and the number of autophagosomes per cell (Fig. 9E, F). These results indicated that ConA treatment led to the blocking of autophagic body degradation in stress-treated microspores. The accumulation of autophagosomes/autophagic bodies after E-64 and ConA treatments also suggested the existence of autophagic flux in microspores after the stress.

**Fig. 10.**
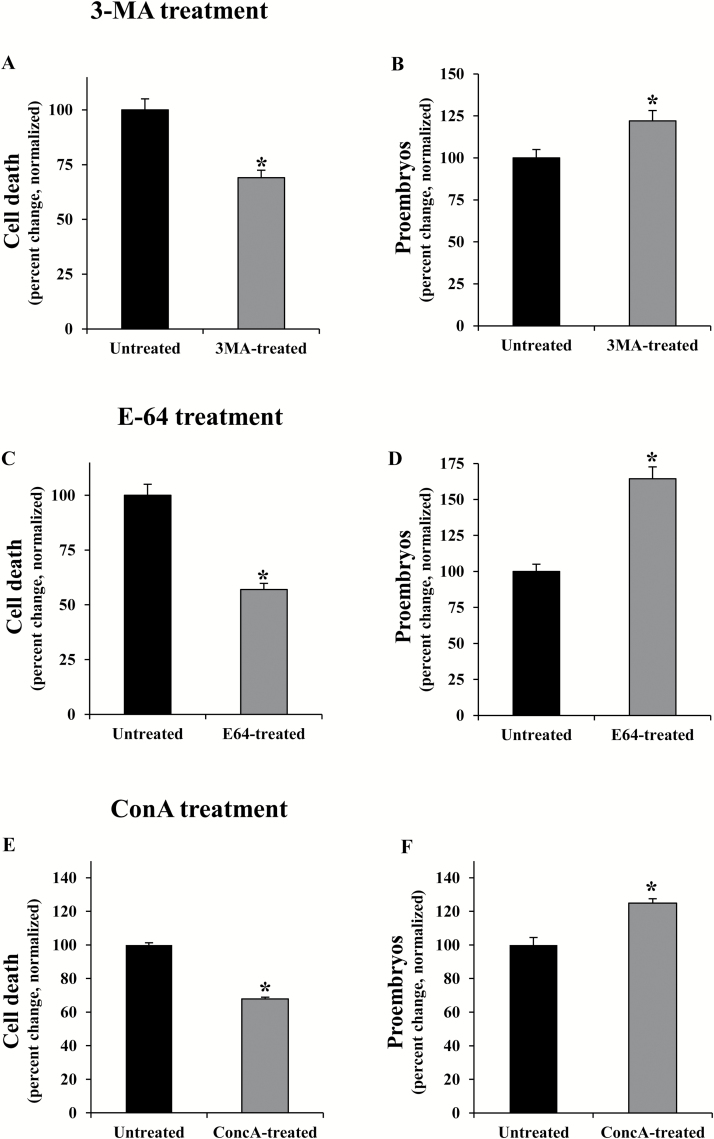
Effects of treatments with 3-MA, E-64, and ConA on cell death and embryogenesis induction in microspore cultures. Quantification of the percentage of dead cells (A, C, E) and proembryos (B, D, F) on microspore cultures 4 d after stress in untreated cultures and cultures treated with 3-MA (A, B), E-64 (C, D), and ConA (E, F). In all histograms, results are expressed as percentages (percent change) and referred to the mean percentage of dead cells or proembryos in untreated cultures which has been normalized to 100%. Bars indicate the SE. Asterisks indicate significant differences between treated and untreated cultures, within each treatment, assessed by Student’s *t*-test, at *P*<0.05.

The effects of the treatments with 3-MA, E-64, and ConA on cell death and embryogenesis induction were also evaluated. After the inductive stress, cell death levels in microspore cultures were significantly reduced by these three inhibitors (Student’s *t*-test, *P*<0.05), in comparison with untreated cultures (Fig. 10A, C, E).

Regarding the quantification of the proembryos in 4 d cultures, the proportion of proembryos in cultures treated with 3-MA, E-64, and ConA was significantly higher than in untreated cultures (Fig. 10B, D, F). These results indicated that inhibition of autophagy by blocking autophagosome formation, with 3-MA, or by inhibition of autophagic body degradation, with E-64 or ConA, improved embryogenesis initiation yield while reducing cell death levels caused by the inductive stress.

### Cathepsin-like activity, gene expression, and subcellular localization in stress-induced microspore embryogenesis

**Fig. 11.**
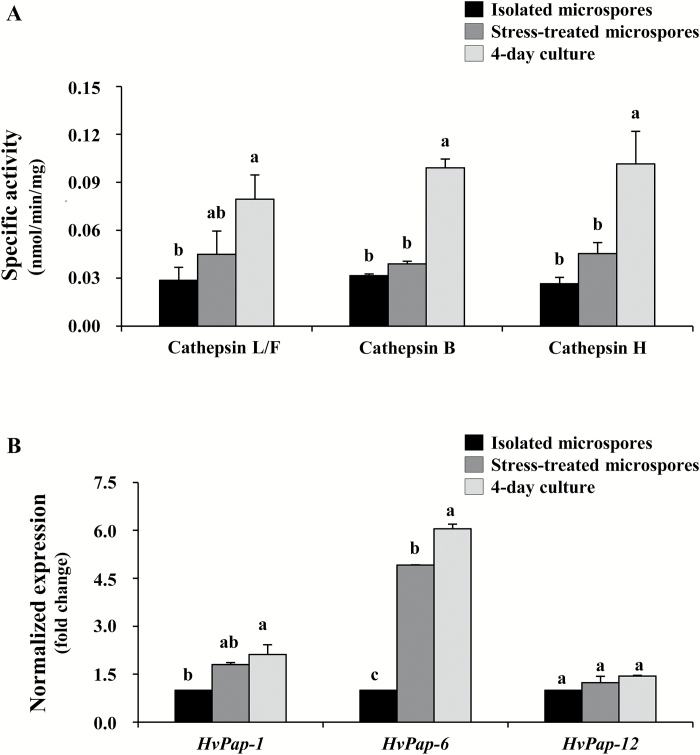
Patterns of cathepsin proteolytic activities and gene expression during stress-induced microspore embryogenesis. (A) Proteolytic pattern of cathepsin L-/F-like, cathepsin B-like, and cathepsin H-like cysteine proteases. Specific activity, in nmol mg^–1^ min^–1^. (B) Transcript levels of the *HvPap-1* gene (cathepsin F-like protease), *HvPap-6* gene (cathepsin L-like protease), and *HvPap-12* gene (cathepsin H-like protease) normalized to the isolated microspore within each gene. Bars indicate the SE. Different letters indicate significant differences among stages within each activity/gene studied, according to ANOVA and Tukey’s tests at *P*<0.05.

We analysed the role of cathepsins in the stress response of microspores because of their relationship with autophagy in animals, and their relevant role as plant cell death proteases. As a first approach, the enzymatic activity was quantified for all the cathepsin activities identified in plants. Significant differences among stages were assessed by ANOVA and Tukey’s tests, with *P*<0.05 conferring statistical significance. Low levels of cathepsin L-/F-, B-, and H-like activities were detected in isolated microspores (Fig. 11A). After the stress, especially in 4 d cultures, cathepsin L-/F-, B-, and H-like proteolytic activities significantly increased, reaching >2-fold the proteolytic values detected before the stress (Fig. 11A).

The expression of several cathepsin genes previously characterized in barley and related to the protease activities detected, *HvPap-1*, *HvPap-6*, and *HvPap-12*, which encode cathepsins of type F-, L-,and H-like, respectively, was also analysed in microspore cultures by RT-qPCR. The three cathepsin genes were expressed at low levels in isolated microspores, while after the stress treatment (in stress-treated microspores and 4 d cultures), *HvPap-1* and *HvPap-6* were induced (Fig. 11B). The cathepsin H-like *HvPap-12* gene did not show significant changes, suggesting that other genes are likely to contribute to the increase of cathepsin H activity detected in microspores after stress. Among the cathepsin genes studied, the cathepsin L-like *HvPap-6* showed the greatest increase in expression after stress, in both stress-treated microspores and 4 d cultures (Fig. 11B).

**Fig. 12.**
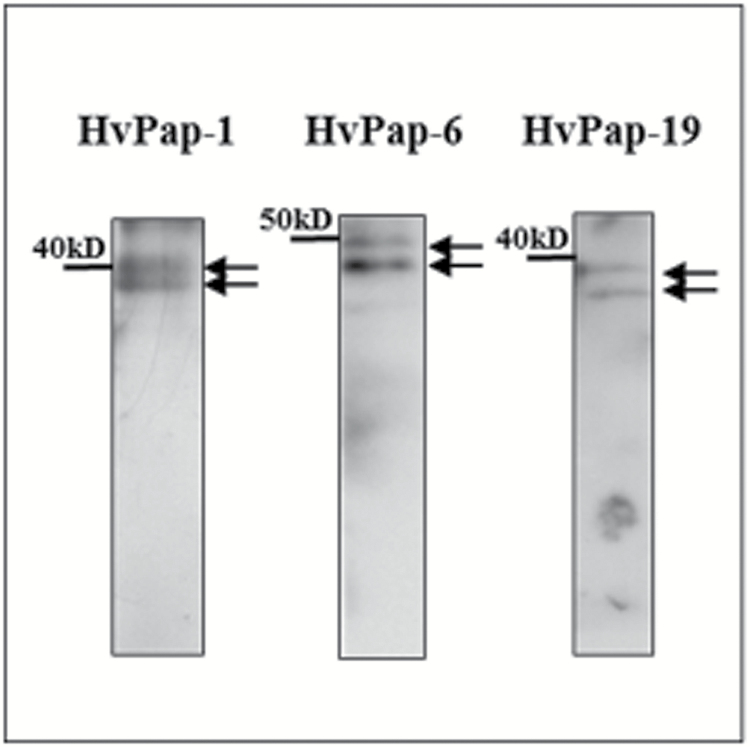
Protein patterns of cathepsins HvPap-1, HvPap-6, and HvPap-19 in microspore-derived embryos detected by immunoblot. Arrows indicate bands corresponding to the inactive (upper) and active (lower) forms of each protease.

To gain more insight into the activation of cathepsins during cell death in stress-induced microspore embryogenesis cultures, the presence and subcellular localization of the proteins HvPap-1, HvPap-6, and HvPap-19 (a cathepsin B-like protein in barley) were analysed using specific antibodies (previously produced in rabbits by Pineda Antibody Services; Díaz-Mendoza *et al.*, 2016). HvPap-12 protein could not be localized since no antibodies were available. The specificity of the antibodies in microspore-derived embryos was assessed by immunoblot assays. Results revealed that each antibody recognized only two bands corresponding to the inactive (zymogen) and active forms of the corresponding C1A protease (Fig. 12). The bands appeared at the expected molecular weights reported for HvPap-1 (40 016 kDa and 26 204 kDa), HvPap-6 (50 226 kDa and 35 158 kDa), and HvPap-19 (37 222 kDa and 29 234 kDa) (Cambra *et al.*, 2012).

**Fig. 13.**
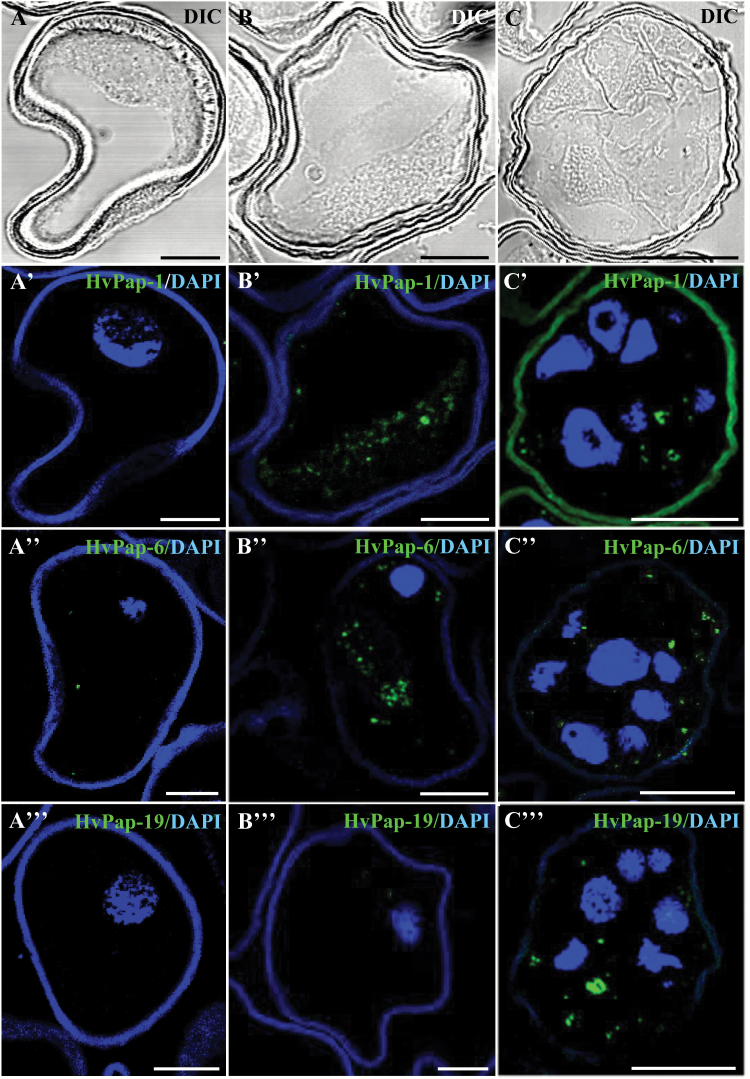
Immunolocalization of barley cysteine proteases (HvPap-1, -6, and -19) during stress-induced microspore embryogenesis. Immunofluorescence and confocal laser scanning microscopy analysis of (A–A''') isolated microspore, (B–B''') stress-treated microspore, and (C–C''') 4 d culture proembryo confined by the exine. (A-C) Normarsky’s differential interference contrast (DIC). (A'–C''') Merged images of cysteine protease immunofluorescence (green) and DAPI staining of nuclei (blue). (A'–C') HvPap-1 cysteine protease. (A''-C'') HvPap-6 cysteine protease. (A'''-C''') HvPap-19 cysteine protease. Scale bars represent in (A–B''') 10 µm, in (C–C''') 20 µm.

Immunofluorescence assays followed by confocal microscopy analyses provided evidence of the induction of cathepsins and their subcellular localization in microspores after the inductive stress to trigger embryogenesis. The results showed very low or no detectable signal with the three cathepsin antibodies on isolated microspores before the stress (Fig. 13A, A', A'', A'''), whereas stress-treated microspores (Fig. 13B, B', B'', B''') and cells of 4 d cultures (Fig. 13C, C', C'', C''') exhibited intense and specific labelling in small cytoplasmic spots of different sizes, probably corresponding to small vacuoles, a pattern that resembled autophagy structures. Patterns of labelling were similar for the three cathepsins, except for HvPap-19, which showed much less labelling in stress-treated microspores (Fig. 13B''') than the others. Controls without the primary antibody did not show any labelling.

## Discussion

### Autophagy is activated and has a role in the cell death promoted by the inductive stress of microspore embryogenesis

The results of the present work provide evidence of the activation and involvement of autophagy in cell death in the response of microspores to the inductive stress triggering embryogenesis in barley. In microspore embryogenesis systems, after the application of the stress treatment, a proportion of the cells present in the *in vitro* culture are reprogrammed, initiating the embryogenesis pathway; these cells are known as responsive cells. Together with the responsive cells, many other cells die, strongly limiting the efficiency of the process (Rodríguez-Serrano *et al.*, 2012).The involvement of plant autophagy in PCD processes during development and pathogen infection is well known (Yang and Bassham, 2015); however, no information had been available until now regarding the role of autophagy in cell death during stress-induced embryogenesis.

Autophagy has been shown to be a rather general response to a variety of abiotic stresses, playing a role in removing damaged proteins and organelles that can be generated as a result of ROS accumulation during oxidative burst. Increasing evidence has connected ROS and autophagy in plants and algae (Pérez-Pérez *et al.*, 2012). In Arabidopsis, it has been demonstrated that oxidative damage caused by ROS generators led to a rapid and strong induction of autophagy (Bassham, 2007). Furthermore, when plants are exposed to abiotic stress conditions, ROS production acts as a common signal to activate stress responses, including autophagy (Bassham, 2009). In a previous study, we reported ROS production in microspores after the inductive stress of embryogenesis, in barley (Rodríguez-Serrano *et al.*, 2012). In the present study, endogenous ROS production has also been detected in barley microspores after stress, while treatments with ROS scavengers lead to a reduction in cell death levels. These results indicate the involvement of these reactive molecules in microspore death in this system.

Furthermore, our results demonstrate that the inductive stress to trigger microspore embryogenesis in barley also induced the activation of autophagy, which is supported by the up-regulation of autophagy *HvATG5* and *HvATG6* genes, the increase of autophagosome-like structures containing ATG5 and ATG8 proteins, and the ultrastructural observation of autophagic structures in microspores after the stress. Moreover, when the inhibitors E-64 or ConA were added to the culture medium, complete autophagosomal degradation is impaired and the proportion of microspores with autophagic structures and their number per cell increased, indicating the active formation of autophagosomes after the stress.

On the other hand, plant proteases with caspase-3-like activity are well known to participate in many PCD processes (Bonneau *et al.*, 2008). In a previous report, we have shown this proteolytic activity to be induced in microspores after the inductive stress to trigger embryogenesis (Rodríguez-Serrano *et al.*, 2012). A recent report has demonstrated that the Arabidopsis cathepsin B protease has caspase-3-like activity and is inhibited by caspase-3-specific inhibitors; AtCathepsin B triple mutants showed a strong reduction in PCD induced by several abiotic stresses, including oxidative stress, indicating a central role for this protease in stress-induced PCD in Arabidopsis (Ge *et al.*, 2016). The results of the present study show the effects of a specific caspase-3 inhibitor, namely the reduction of cell death levels in microspore cultures, providing additional evidence for the involvement of caspase-like activities in the stress-induced cell death in microspores. In barley caryopsis, a VEIDase protease was found to have a caspase-like activity; it was localized to autophagosomes, linking the caspase activity to autophagic PCD (Borén *et al.*, 2006). The results presented here show that autophagy and cell death are also connected to caspase-3-like proteolytic activity in microspores treated with the inductive stress of embryogenesis.

The role of autophagy in degradation of cellular components during PCD execution has been reported in various plant PCD processes during development, such as suspensor degradation in spruce somatic embryogenesis (Minina *et al.*, 2013, 2014*a*, *b*), ovary degradation in wheat, petal senescence, and xylogenesis (reviewed in Bassham, 2009). Autophagy has also been implicated in PCD induced by pathogens and other injuries (Hofius *et al.*, 2011, 2017). New results are reported here on the activation of autophagy associated with cell death occurrence, in response to the inductive stress triggering microspore embryogenesis. Nevertheless, the exact role of autophagy in cell death is still not completely understood. The functional analyses performed in the present study with several autophagy inhibitors have revealed the implication of autophagy in cell death occurrence. The results presented here reveal that 3-MA inhibited autophagy in stress-treated microspores of barley, as in other plant systems, most probably impairing autophagosome formation (Takatsuka *et al.*, 2004). Moreover, 3-MA treatment resulted in the reduction of cell death levels of microspores after stress. Secondary effects of 3-MA have been reported in some systems, such as in Arabidopsis root hairs, where 3-MA could inhibit mitochondrial-activated PCD rather than autophagy (Kacprzyk *et al.*, 2014). Although possible secondary effects of 3-MA in microspores cannot be completely ruled out, there is no evidence in microspores of mitochondrial activation of PCD, and our results indicate that in microspores 3-MA inhibits autophagy and leads to a reduction of cell death. Treatments with other autophagy inhibitors, such as E-64 and ConA, which block autophagosome degradation, have been used in plant tissues and suspension cells (Matsuoka *et al.*, 1997; Sláviková *et al.*, 2005; Moriyasu and Inoue, 2008; Shin *et al.*, 2014; Bassham, 2015; Yano *et al.*, 2015). Their application in microspore cultures also leads to impaired autophagy activity in microspores after the stress, and to a decrease in cell death levels. Therefore, these results indicate the involvement of autophagy, at least in part, in the death of the microspores in response to the stress.

Autophagy is activated in response to many physiological cues and stress conditions, and has been associated with both cell survival and cell death. Depending on the context and intensity, autophagy can protect cells or mediate cell death (Kroemer and Jäättelä, 2005). In the case of microspore embryogenesis, the response of microspores to the stress treatment depends on many factors, such as the physiological state and the developmental stage of the cell. Only a certain proportion of the heterogeneous cell population of the microspore cultures tolerates the stress and is responsive to embryogenesis induction, whereas many other cells cannot tolerate the stress and die. Our results show that the application of the inductive stress leads to the activation of autophagy that plays a role in the death of cells, since autophagy inhibition reduces cell death levels. As a consequence of this reduction in cell death, embryogenesis induction was enhanced. On the other hand, the possibility that autophagy activity could also have a prosurvival function in some other stress-treated cells cannot be completely ruled out.

### Together with autophagy, cathepsins are induced and participate in the cell death of microspores after the inductive stress of embryogenesis

Because cathepsins are well known lysosomal proteases with a role in autophagy and cell death, in animals (Turk and Stoka, 2007), and as they are major proteases with reported functions in cell death also in plants, we have analysed the participation of cathepsins in the microspore response to the inductive stress. In animals, numerous reports have documented the critical role of cathepsins in the degradation of cytoplasmic organelles and components through autophagy, being responsible for the terminal degradation of proteins within autolysosomes (Kroemer and Jäättelä, 2005; Jung *et al.*, 2015; Man and Kanneganti, 2016). Nevertheless, much less information is available on plant cathepsins.

The results of our study revealed the participation of C1A proteases (cathepsins) in stress-induced microspore embryogenesis, with the up-regulation of cathepsin genes *HvPap-1* and *HvPap-6*, which encode cathepsins F- and L-like, respectively, after the stress. Concomitantly, the cathepsin L-/F-, B-, and H-like proteolytic activities increase in stress-treated microspores, as does the presence in their cytoplasm of proteins HvPap-1, HvPap-6, and HvPap-19 (a cathepsin B-like protein in barley; Díaz-Mendoza *et al.* 2016). These proteases localized in small cytoplasmic spots of various sizes, probably corresponding to vesicles, lysosomal-like organelles, and small vacuoles of stress-treated microspores, a localization pattern that resembles that of autophagy structures. These results indicate the role of cysteine C1A proteases in the microspore response to stress.

In barley, C1A proteases, specifically HvPap-1, have been reported to participate in the proteolysis induced in leaves by abiotic stresses such as darkness and nitrogen starvation (Velasco-Arroyo *et al.*, 2016), as well as in the development and germination of barley grains (Díaz-Mendoza *et al.*, 2016). Nevertheless, the role of HvPap-1 in PCD has not been previously described. Likewise, little is known about the function of HvPap-6 and HvPap-19 in this process. In Arabidopsis, Zhang *et al.* (2014) reported that CEP1, a C1A cysteine protease, plays a key role in tapetal PCD, a process that critically regulates pollen development. Our results demonstrate that these proteases contribute to the response to stress of microspores. Moreover, when we treated microspores with E-64, which inhibits intracellular cysteine proteases, the levels of cell death decreased, suggesting the involvement of these proteases in cell death in stress-treated microspore cultures. As a consequence of this reduction in cell death, embryogenesis induction was enhanced, which opens up new possibilities for biotechnological manipulation of the process with cysteine protease modulators to improve the yield of *in vitro* embryogenesis systems.

Several reports in animals have demonstrated that treatment with inhibitors of the lysosomal cysteine proteases, such as cathepsins B- and L-like proteases, impairs autophagy, and mutants with reduced cathepsins B and D show impaired autophagic degradation (Tatti *et al.*, 2012). A previous study connected autophagy and cathepsins to the promotion of cell death associated with the hypersensitive response to pathogens in Arabidopsis (Hofius *et al.*, 2009), and it was suggested that they could contribute to different cell death pathways operating in plant immunity responses. The pattern of localization of HvPap cathepsins in microspores as cytoplasmic spots of different sizes is consistent with them being located in vesicles, lysosomal-like structures and small vacuoles, some of which could represent autolysosome-like structures and small autophagic vacuoles. This fact, together with the activation of both autophagy and cathepsins after the stress, the similar induction of cathepsin and ATG gene expression after the stress, and the observation of the same effects in reducing cell death by the inhibition of both actions, suggests a connection between C1A proteases (cathepsins) and autophagy in stress-treated microspores, as has been widely demonstrated in mammalian cells (Man and Kanneganti, 2016), although further work will be required to prove this connection. The induction of autophagy after the stress, together with the activation of cathepsins, may be crucial in the orchestration of cell death among other cell responses to the inductive stress, therefore participating in the control of success of embryogenesis initiation.

### Conclusions

In summary, the results reported here reveal that autophagy is activated after the inductive stress used to trigger microspore embryogenesis in barley, and its pharmacological inhibition reduces cell death levels, indicating a role for autophagy in the stress-induced cell death of microspores. Cathepsin protease activities are concomitantly induced, and their inhibition also impaired cell death. The similar patterns of activation, expression, and localization of autophagy and cathepsins suggest a connection between both activities in stress-induced cell death during microspore embryogenesis induction, a hypothesis that needs further analyses. The findings provide new insights into the mechanisms underlying the microspore response to the inductive stress, opening up new possibilities to enhance microspore embryogenesis efficiency in recalcitrant species while reducing cell death levels with modulators of autophagy and cysteine proteases.

## Supplementary data

Supplementary data are available at *JXB* online.

**Table S1.** Cathepsin-like protease amino acid sequences (peptides) used for specific antibody production.

**Table S2.** Primer sequences used for the amplification of genes by RT-qPCR assays.

**Method S1.** Methods for the production of ATG5 antibodies with the recombinant ATG5 protein of *Picea abies*.

Supplementary Table S1-S2

Supplementary Method S1

## Abbreviations:

ATG: autophagy-related protein
3-MA: 3-methyladenine
MDC: monodansylcadaverine
PCD: programmed cell death
ROS: reactive oxygen species.
