# Supplementary material for: Autophagy is activated and involved in cell death with participation of cathepsins during stress-induced microspore embryogenesis in barley
Source: J Exp Bot. 2018 Jan 4;69(6):1387–402. doi: 10.1093/jxb/erx455 (PMC6019037; doi:10.1093/jxb/erx455)
Supplement: Supplementary Table S1-S2 [file erx455_suppl_supplementary_table_s1-s2.pdf]

Table S1. Cathepsin-like protease amino acid sequences (peptides) used for specific antibody production.

Barley proteases	Peptides
HvPap-1	SGFAPSRFKEKPYWIIKN
HvPap-6	IDSEEDYPYKERDNRC
HvPap-19	CQEKKHFSIDAYQVNSDPHD

Table S2. Primer sequences used for the amplification of barley genes by RT-qPCR assays. *HvCycl* gene (cyclophilin), *HvPap-1* gene (cathepsin F-like protease) *HvPap-6* gene (cathepsin L-like protease), *HvPap-12* gene (cathepsin H-like protease), autophagy related genes *HvATG5* and *HvATG6*.

**Barley genes**	**Primers sequence**	
	**Forward**	**Reverse**
HvCycl	TCCACCGGAGAGGAAGTACAGT	AATGTGCTCAGAGATGCAAGGA
HvPap-1	TCCTGGAGTCGATCTTTGGTTTC	CAAGCATACTGTTGCGGCTTC
HvPap-6	TGCAATTGACGGCAAGAAGA	TGGATCACCAGGTGATCATTTG
HvPap-12	ATGTGCGCTATTGCTACCTGC	CACCTTATTCATGTCTGGCGAA
HvATG5	TGGGAATCAGTGAGGAAAGG	CCAGGAGATTCAGGCTCATC
HvATG6	TGGTTTCTCACTTGCCTTCA	AGCCTTTGTCCAGTTCTCCA
