# Supplementary material for: Autophagy is activated and involved in cell death with participation of cathepsins during stress-induced microspore embryogenesis in barley
Source: J Exp Bot. 2018 Jan 4;69(6):1387–402. doi: 10.1093/jxb/erx455 (PMC6019037; doi:10.1093/jxb/erx455)
Supplement: Supplementary Method S1 [file erx455_suppl_supplementary_method_s1.pdf]

Method S1: Methods for the production of antibodies to the recombinant ATG5 protein of *Picea abies*.

ATG5-Pa recombinant protein production and purification. ATG5-Pa (GeneBank accession number HE793992.1.) cDNA was subcloned into expression vector pET28a, and recombinant protein was expressed in *Escherichia coli* BL21-codonPlus-(DE3)- RIL strain (Stratagene). Bacterial cells were grown at 37 ºC of Luria–Bertani broth supplemented with 50 μg mL^{-1} kanamycin and 20 μg mL^{-1} chloramphenicol until DO_{600} reached 0.6. For induction, isopropyl-β-d-thiogalactoside (IPTG) was added to a final concentration of 1 mM, being the culture supplemented with 25 μg mL^{-1} kanamycin and 10 μg mL^{-1} chloramphenicol. After 3 hours expression at 28 ºC, cells were harvested by centrifugation at 15,000 xg for 5 min and resuspended in extraction buffer (50 mM NaH_2PO_4, pH 7, 300 mM NaCl, 8 M urea and 10 mM imidazole). Cells were sonicated (Branson Sonifier 250) and cell debris pelleted down by centrifugation at 16,000 xg. Supernatant was filtered through 0.2 μm nitrocellulose membranes and incubated in Ni-NTA agarose (Qiagen) for 20 minutes. Washing buffers composition was 50 mM NaH_2PO_4, pH 7,300 mM NaCl, 6 M urea. In total, seven washes of the resin were performed: three washes were made supplementing wash buffer with 20 mM imidazole, three ones with 40 mM and one last wash with 60 mM imidazole. Wash buffer containing 250 mM of imidazole was used for protein elution. Purified recombinant protein was dialyzed against 50 mM Hepes pH 6.8, 2 mM EGTA, 2 mM Mg_2SO_4, 2 mM DTT, 50 mM $CaCl_2$ and 10% (v/v) glycerol (Slide-A-Lyzer® Dialysis Kit 3,500 MWCO from Thermo Scientific).

Antibodies production and purification. Polyclonal antiserum against 500 μg of spruce purified recombinant ATG5 was raised in New Zealand white rabbits (Davids Biotechnologie GmbH). Purified antigen was blotted onto a PVDF membrane by standard electrophoretic transfer and incubated with the immune serum. Enriched polyclonal antibodies were then eluted from the membrane with 0.2 M Glycine pH 2.2, equilibrated with Tris-HCl pH 8.8 and stored at -80C.
